# Use of reliable contraceptives and its correlates among women participating in Simulated HIV vaccine efficacy trials in key-populations in Uganda

**DOI:** 10.1038/s41598-019-51879-2

**Published:** 2019-10-28

**Authors:** Andrew Abaasa, Jim Todd, Yunia Mayanja, Matt Price, Patricia E. Fast, Pontiano Kaleebu, Stephen Nash

**Affiliations:** 1 MRC/UVRI & LSHTM Uganda Research Unit, Entebbe, Uganda; 2 London School of Hygiene and Tropical Medicine, London, UK; 3 International AIDS Vaccine Initiative, New York, USA; 4 University of California at San Francisco, Department of Epidemiology and Biostatistics, San Francisco, USA; 5 Pediatric Infectious Diseases, School of Medicine, Stanford University, Palo Alto, California, USA

**Keywords:** Epidemiology, Disease prevention, Phase III trials, Epidemiology

## Abstract

To prevent pregnancy in trials, reliable contraceptive use is key. We investigated reliable contraceptive use at baseline and six months in key-populations in Uganda, during two Simulated HIV Vaccine Efficacy trials (SiVETs). SiVETs were nested within observational cohorts of Fisherfolk (2012–2014) and Female sex workers (2014–2017). Women in the observational cohorts were screened and enrolled into the SiVET. The trial administered a licensed Hepatitis B vaccine at 0, 1 and 6 months. Contraceptive use data were recorded at baseline and follow-up clinic visits. Reliable contraceptives (injectable Depot Medroxyprogesterone Acetate (DMPA), implant, pills, and intrauterine device (IUD)) were promoted and provided to women not using a reliable method at enrolment. Overall, 367 women were enrolled. At baseline 203 (55%) reported use of reliable contraceptive. Of the 164 women not using a reliable method at enrolment, 131 (80%) started using them during follow-up bringing the overall number to 334 (91%) at the end of follow-up. Young age (≤35 years) was an independent predictor of reliable contraceptive use at both time points while other factors varied. Promotion and provision of reliable contraceptives increased the proportion using them and could help reduce the risk of pregnancy in future HIV prevention trials.

## Introduction

Sub-Saharan Africa (SSA) suffers the highest burden of HIV with 70% of the people living with HIV in 2017 being residents in SSA^1^. Similarly, global estimates show that 65% of new HIV infections in 2017 happened in this region^1^. Some sub-populations, such as members of fishing communities (fisherfolks-FF) and female sex workers (FSW), are disproportionately affected. The HIV prevalence in fishing communities is as high as 37%^2–6^ and annual HIV incidence of more than 3 per 100 person years have been reported^7,8^. The HIV burden is worse among women^2,4^. Because of the high HIV incidence, these communities are attractive for the conduct of HIV vaccine efficacy trials. However, trials could take months or years from recruitment to completion. In this long period, women could become pregnant and might have to be withdrawn from follow up due to unknown effects of the new investigational product on the fetus. More withdrawals than that anticipated could have negative effects on the trial statistical power^9–11^. In such efficacy trials, it is important to prevent pregnancies in women participants through the use of reliable, long-acting, reversible contraceptive methods.

Reliable contraceptives defined as non-barrier methods likely to reduce the risk of pregnancy include injectable Depot medroxyprogesterone acetate (DMPA), pills, Norplant-implant and intrauterine contraceptive device (IUCD)^12^. The use of reliable contraceptives in women of reproductive age is low, at 64% globally, 28% in SSA and 40% in East Africa^13^. Lack of access to and concerns regarding side effects or health risks associated with contraceptives use have been the main reasons advanced for the low use in SSA^14^. In Uganda, 35% of women in the general population use reliable contraceptives, although this may be higher in specific sub-groups of the population^15^.

Previous HIV prevention trials in non-fishing communities in Africa (East, West and Southern Africa) have shown that reliable contraceptive use is low, ranging between 5% to 28%^12,16–19^. In a 2013 review of microbicide trials in Africa the incidence of pregnancy ranged from 4.0 to 64 per 100 women-years^20^. There is little data on uptake of reliable contraception and the impact of contraception on pregnancy during these trials.

High levels of willingness (>95%) to participate in HIV vaccine efficacy trials have been shown in Africa^21,22^. However, among women this decreased to 23% when the need to prevent or delay pregnancy through use of reliable contraceptives was mentioned^21^. To date, no HIV vaccine efficacy trials have been conducted in women at high-risk of HIV infection in Uganda, and there is no baseline information on the use of reliable contraceptive methods to delay pregnancy during HIV prevention trials. It is unknown how the use of reliable contraceptives and risk of pregnancy would change among study participants and how this would affect HIV vaccine efficacy trials in these populations.

In populations where no baseline data on reliable contraceptives use is available, prospective trial participants may be required to use reliable contraceptive methods for at least three months before screening and enrolment^23^. This increases the cost of conducting trials and delays rollout, but avoids costly drop out from trials due to non-compliance. Studies aimed at establishing factors associated with reliable contraceptives use under efficacy trial conditions, can provide baseline data to be used in planning such trials in the FF and FSW populations where little or no information is available.

Recently, we conducted two “Simulated HIV Vaccine Efficacy Trials (SiVETs)” in which procedures and criteria for the trial mimicked an HIV vaccine efficacy trial, but the vaccine used was a licensed Hepatitis B vaccine. Participants were informed that this trial was a simulation and the vaccine would protect them against Hepatitis B, not HIV. The SiVET concept is suggested to be useful in assessing the feasibility for conduct of clinical trials of a new product, through a “simulated” trial using a commercially available vaccine^24,25^. This concept can inform the design and sample size estimation for the future trials^26–28^.

In this paper, we use data from the two SiVETs, in which reliable contraceptives were promoted and provided at no cost to female participants, to: determine the proportion of women using a reliable method (i) at baseline and (ii) at the end of vaccination schedule (6 months of follow up), and to determine the correlates of reliable contraceptives use (iii) at baseline and (iv) at 6 months of follow up.

## Methods

The data for this paper come from two Simulated HIV Vaccine Efficacy Trials (SiVETs) in Uganda. To assess and improve readiness for efficacy trials of HIV preventive vaccines or other investigational agents among key populations in Uganda, we conducted two trials in which licensed Hepatitis B vaccine was used in a protocol that otherwise resembled an efficacy trial for HIV vaccine. These were nested in, respectively, an observational cohort of FF (Jul 2012–Apr 2014) in Masaka and a cohort of FSW (Aug 2014–Apr 2017) in Kampala. Both studies were conducted by MRC/UVRI & LSHTM Uganda Research Unit clinics supported by the International AIDS Vaccine Initiative (IAVI). The observational cohorts details have been previously described^6,8,29,30^. Sexually active (self-reported having sex in the preceding three months) HIV negative women who had been part of the observational cohorts’ quarterly follow up (aimed at determining HIV incidence) for between three and 18 months were screened for eligibility (Table 1) for enrollment into the SiVET. Those eligible were asked if they were using any method of contraceptive. The contraceptive methods were classified as either reliable (injectable DMPA, implant, oral pills, and IUD), or unreliable (condoms, lactational amenorrhea, withdrawal etc.). The study nurse promoted reliable contraceptives to women who were not using any method, or were using an unreliable method, at baseline. Eligible women who were either already using a reliable method or were willing to initiate one were enrolled into the SiVET. They were provided with a reliable contraceptive method of their choice. While DMPA and oral pills were provided at both study site clinics (Kampala and Masaka), implant and IUD were provided by the study staff only at the Kampala clinic. At the Masaka clinic, women were referred to a Marie Stopes clinic located about one kilometre from the study research site clinic where they could obtain implant or IUD.

**Table 1:** Eligibility Criteria for screening/enrolment into SiVET at Kampala & Masaka clinics, Uganda.

Eligible	Ineligible
• At least 3 and no more than 18 months of follow up in the observational cohort	• HIV-1 infection
• HIV-1 negative and willing to undergo HIV testing	• Pregnant or intending to get pregnant
• Aged ≥18 years and ≤49 years	• Previous exposure to Hepatitis B or current infection (only Kampala clinic)
• Able and willing to provide informed consent	• History of severe allergic reaction to any substance
• Able and willing to provide adequate locator information	• An acute or chronic illness
• Willing and able to return for follow-up clinic visits	• Contraindication for Hepatitis B vaccine
• Intending to reside in the study area for at least one year	• Participation in another clinical trial
• Willing to undergo pregnancy testing	
• Not breastfeeding and no intent for pregnancy in the next year	
• Willing to use a reliable method of contraceptives during the study	

All enrolled women were provided with a contraceptive card, which captured the method they were using and any future changes or renewals. They were requested to carry their card every time they visited the study research clinic. Contraceptive use data was collected at enrolment, and at months one, three, and six. At enrolment, women were administered a licensed Hepatitis B vaccine (ENGERIX-BTMGlaxoSmithKline Biologicals Rixensart, Belgium) following the standard schedule of 0, 1 and 6 months, akin to what might happen in an HIV vaccine efficacy trial. After each vaccination, women were retained in the clinic for at least 30 minutes for reactogenicity events assessment. Furthermore, they were requested to return after three days for further review. Details of the trial procedures and follow up have been previously described^31,32^.

Pregnancy testing: Women were asked to provide a urine sample at each clinic visit for pregnancy testing. A QuickVue One-Step human chorionic gonadotropin (HCG) test (manufacturer: Quidel Q20109IN) was used to determine pregnancy.

### Statistical analysis

SiVET data were captured using OpenClinica 3.5 **(**Waltham, MA). The data were transferred to Stata 14 (StataCorp, College Station, TX USA) for cleaning and analysis. Variables examined included; social demographics- age, tribe, education, religion, marital status, occupation and duration of residency. Behavioral - frequency of alcohol consumption, having sex under the influence of alcohol, illicit drug use, number of sexual partners, having a new sexual partner other than the regular partner, frequency of condom use with a new sexual partner and being away from home for at least 3 days per week. Clinical – having had a genital discharge and/or genital sores/ulcer disease in the three months preceding the interview. Participant baseline socio-demographic, study site, HIV risk behavior, and clinical characteristics were summarized using counts and percentages and further stratified by study population and whether or not a participant reported use of a reliable contraceptive method at enrolment. Similarly, participant characteristics were compared between those who took up a reliable contraceptive method and those who did not. The numbers of women who took up a reliable contraceptive method overall, and at each clinic visit, were presented using a bar graph. We defined uptake of reliable contraceptives as a woman using an unreliable or no method at enrolment into SiVET but taking up one of the reliable methods at any one point during follow up. Simple logistic regression models were fitted to determine correlates of reliable contraceptives use at baseline and at the last vaccination visit (six months of follow up). After bivariable analyses, a multivariable model was fitted. In the multivariable model, factors were removed from the model using a backward elimination algorithm retaining any factors, which caused a change in the log odds of 20% or more.

## Results

### Screening

**Figure 1:**
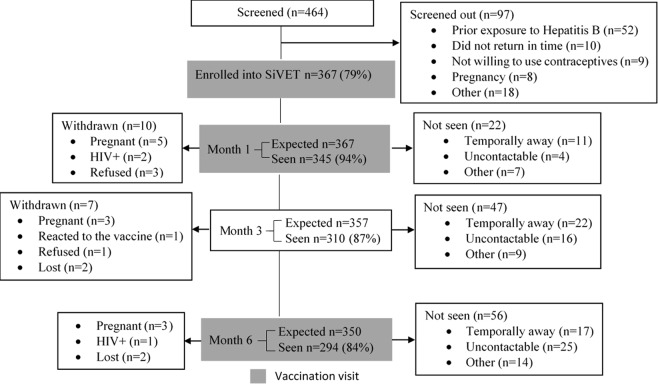
Study profile of screening, enrolment and follow up of 367 participants in SiVET at Kampala and Masaka clinics, Uganda (2012–2017).

In total, 464 [FF = 83 and FSW = 381] sexually active women were screened for entry into the SiVETs, of whom 367 (79%; FF = 77 (93%) and FSW = 290 (76%)) non-pregnant women were enrolled, overall screening-enrolment ratio of 5:4. Of the 97 women ineligible for enrolment, the primary reason was prior exposure to Hepatitis B (54%, n = 52). Eight women (8%) were excluded because of pregnancy; other reasons for exclusion are shown in Fig. 1.

### Baseline characteristics

In the FF population, the average age of enrolled women was 30 years (SD = 7.5, range 18–49), with 38 (49%) being of the indigenous Baganda tribe, 57 (74%) of Christian faith, 49 (64%) had primary education and 54 (70%) had lived at the current location for more than one year. All participants reported having a new sexual partner (not a regular sexual partner) in the three months preceding the interview and 47 (61%) reported never using a condom while having sex with the new sexual partner (Table 2). In the FSW population, the average age was 28 years (SD = 7.5, range 18–49), with 153 (53%) being of the indigenous Baganda tribe, 219 (76%) of Christian faith, 149 (51%) had primary education, 204 (70%) single but previously married. Two hundred and thirty nine (82%) had lived at the current location for more than one year. A total of 235 (81%) reported illicit drug use, 287 (99%) reported two or more sexual partners, 266 (92%) reported having a new sexual partner in the 3 months preceding the interview and 207 (78%) reported that they always use a condom while having sex with a new sexual partner (Table 2).

**Table 2:** Participant baseline characteristics, proportion and factors associated with using reliable contraceptives among 367 participants enrolled in SiVET in Uganda (2012–2017).

Participant characteristic	FF n (%)	FSW n (%)	Contraceptive use		uOR (95%CI)	LRT-pvalue	aOR (95%CI)
			FF	FSW			
Overall	77 (100)	290 (100)	24 (31.2)	179 (61.7)			
Site						<0.001	
Masaka	77 (100)	na	24 (31.2)	na	1.00		1.00
Kampala	na	290 (100)	na	179 (61.7)	3.56 (2.08–6.09)		1.56 (0.66–3.69)
Age group (years)						0.001	
35+	21 (27.3)	62 (21.4)	5 (23.8)	28 (45.2)	1.00		1.00
18–34	56 (72.7)	228 (78.6)	19 (33.9)	151 (66.2)	2.26 (1.37–3.72)		2.07 (1.21–3.54)
Tribe						0.107	
Muganda	38 (49.3)	153 (52.8)	12 (31.6)	98 (64.1)	1.00		
Munyankole	7 (9.1)	32 (11.0)	2 (28.6)	19 (59.4)	0.86 (0.43–1.72)		
Munyarwanda	18 (23.4)	20 (6.9)	4 (22.2)	10 (50.0)	0.43 (0.21–0.88)		
Other	14 (18.2)	85 (29.3)	6 (42.9)	52 (61.2)	1.04 (0.64–1.70)		
Education						0.183	
None	7 (9.1)	16 (5.5)	3 (42.9)	10 (62.5)	1.0		1.00
Primary	49 (63.6)	149 (51.4)	14 (28.6)	87 (58.4)	0.80 (0.34–1.91)		0.81 (0.32–2.05)
Secondary+	21 (27.3)	125 (43.1)	7 (33.3)	82 (65.6)	1.20 (0.49–2.92)		1.00 (0.38–2.61)
Religion						0.685	
Christian	57 (74.0)	219 (75.5)	20 (35.1)	131 (59.8)	1.00		
Muslim	20 (26.0)	71 (24.5)	4 (20.0)	48 (67.6)	1.10 (0.68–1.78)		
Marital status						0.319	
Single, never married	15 (19.5)	68 (23.5)	7 (46.7)	38 (55.9)	1.00		
Married	30 (39.0)	18 (6.2)	11 (36.6)	11 (61.1)	0.71 (0.35–1.46)		
Single, previously married	32 (41.5)	204 (70.3)	6 (18.7)	130 (63.7)	1.15 (0.77–1.82)		
Occupation						0.002	
Fishing/related	21 (27.3)	0 (0.0)	5 (23.8)	0 (0.0)	1.00		
Small scale business	27 (35.1)	11 (3.8)	6 (22.2)	9 (81.8)	2.09 (0.63–6.90)		
Hotel/Bar/Saloon	19 (24.6)	111 (38.3)	10 (52.6)	67 (60.4)	4.65 (1.61–13.46)		
Sex work	0 (0.0)	165 (56.9)	0 (0.0)	101 (61.2)	5.05 (1.76–14.46)		
Other	10 (13.0)	3 (1.0)	3 (30.0)	2 (66.7)	2.00 (0.45–8.98)		
Duration lived at current location						0.614	
0–1, year	23 (29.9)	51 (15.6)	8 (34.8)	31 (60.8)	1.00		
>one year	54 (70.1)	239 (82.4)	16 (29.6)	148 (61.9)	1.14 (0.68–1.90)		
Alcohol consumption (previous one month)						0.099	
None	33 (42.9)	57 (19.7)	11 (33.3)	30 (52.6)	1.00		
Inconsistent	38 (49.3)	147 (50.6)	11 (29.0)	98 (66.7)	1.71 (1.03–2.85)		
Daily	6 (7.8)	86 (29.7)	2 (33.3)	51 (59.3)	1.62 (0.90–2.92)		
Sex under alcohol influence (previous one month)						0.222	
Never	45 (58.4)	103 (35.5)	12 (26.7)	63 (61.2)	1.00		
Sometimes	24 (31.2)	169 (58.3)	10 (41.7)	105 (62.1)	1.44 (0.93–2.21)		
Frequently	8 (10.4)	18 (6.2)	2 (25.0)	11 (61.1)	0.97 (0.42–2.24)		
Drug use (previous one month)						<0.001	
No	71 (92.2)	55 (19.0)	21 (29.6)	24 (43.6)	1.00		1.00
Yes	6 (7.8)	235 (81.0)	3 (50.0)	155 (66.0)	3.43 (2.18–5.38)		2.45 (1.38–4.35)
Genital discharge (previous 3 months)						0.055	
Yes	51 (55.2)	88 (30.3)	14 (27.4)	54 (61.4)	1.00		
No	26 (33.8)	202 (69.7)	10 (38.5)	125 (61.9)	1.52 (0.99–2.31)		
Genital sores/ulcer disease (previous 3 months)						0.009	
Yes	40 (52.0)	48 (16.6)	11 (27.5)	27 (56.3)	1.00		
No	37 (48.0)	242 (83.4)	13 (35.1)	152 (62.8)	1.90 (1.17–3.09)		
Number of sexual partners (previous 3 months)						<0.001	
0/1	47 (61.0)	3 (1.0)	13 (27.7)	2 (66.7)	1.00		1.00
2+	30 (39.0)	287 (99.0)	11 (36.7)	177 (61.7)	3.40 (1.78–6.48)		1.30 (0.52–3.29)
New sexual partner (previous 3 months)						0.758	
No	0 (0.0)	24 (8.3)	0 (0.0)	14 (58.3)	1.00		
Yes	77 (100)	266 (91.7)	24 (31.2)	165 (62.0)	0.88 (0.38–2.03)		
Condom use with the new sexual partner						0.001	
Never	47 (61.0)	9 (3.4)	15 (31.9)	6 (66.7)	1.00		
Sometimes	19 (24.7)	50 (18.8)	6 (31.6)	26 (52.0)	1.44 (0.70–2.96)		
Always	11 (14.3)	207 (77.8)	3 (27.3)	133 (64.3)	2.76 (1.51–5.07)		
Away from home ≥3 days/week						0.741	
Yes	24 (31.2)	98 (33.8)	6 (25.0)	60 (61.2)	1.00		
No	53 (68.8)	192 (66.2)	18 (34.0)	119 (62.0)	1.08 (0.70–1.67)		

uOR-Unadjusted odds ratio, aOR-Adjusted odds ratio, CI-Confidence interval, %-percent, na-Not applicable, LRT = likelihood ratio test. FF-Fisherfolk, FSW-Female sex workers.

### Baseline contraceptives use (Table 2)

Of the 367 women enrolled, 213 (58%; FF = 30 (39%) and FSW = 183 (63%)) reported use of some form of contraceptive at baseline. Reliable methods were used by 203 women (55%; FF = 24 (31%) and FSW = 179 (62%)) which included 136 (67%) women using injectable DMPA, 30 (14.8%) using an implant, 29 (14%) using oral pills, 6 (3%) using an IUD and 2 (1%) women sterilized. A further 9 (3%) women used condoms and one woman (0.3%) used lactational amenorrhea. No reasons were documented for the 154 women not using contraceptives. In total, 164 women (10 using unreliable and 154 not using any method) were not using any reliable contraceptives at baseline. Adjusting for factors indicated in Table 2, age and self-reported illicit drug use were independently associated with reliable contraceptives use at baseline. Women aged 18–34 years were twice as likely to use a reliable method, adjusted odds ratio (aOR) = 2.07, 95%CI: 1.21–3.54 compared to those aged 35 years or more. Similarly, self-reported illicit drug users were twice more likely to use reliable contraceptives aOR = 2.45, 95%CI: 1.38–4.35 compared to non-illicit drug users (Table 2).

### Retention

In total, 294 (84% of 350 expected and 80% of 367 enrolled; FF = 64/71 (90%) and FSW = 230/279 (82%)) completed all four study visits, up to six months. Overall, 23 (6%) were withdrawn from the study over the six months follow up, 11 (3%) due to pregnancy, and 12 (3%) for other reasons (see Fig. 1). Of the 11 women who became pregnant, seven (four on injectable and three on oral pills) reported using reliable contraceptive before the pregnancy while four were not using any method. The four on injectable had delayed injection by 25, 27, 35 and 40 days. Of the 367, 357 and 350 women expected at month one, three and six respectively, 94%, 87% and 84% were seen. The reasons for missing a given visit are indicated in Fig. 1. Compared to those who completed all the study follow up visits, those that missed any one visit were younger (mean age, 26.3 vs. 29.7 years), with one or no sexual partner 44% vs two or more 29%, new sexual partner 42% vs none 31%, spent up to one year at the current location 41% vs more than one year 29%.

### Uptake of reliable contraceptives

**Figure 2:**
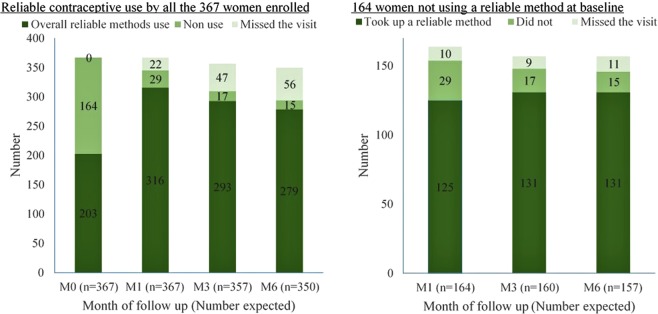
Reliable contraceptives use overall and uptake by the 164 women that were not using a reliable method at enrolment into SiVET, Uganda (2012–2017).

The trial promoted reliable contraceptive methods to the 164 women (FF = 53 and FSW = 111) not using a reliable method at baseline; of these women 131 (80%; FF = 39 (74%) and FSW = 92 (83%)) reported using a reliable contraceptive method during at least one follow up visit. Figure 2 shows the number of women that used reliable contraceptives at each of the follow up visits, throughout the trial. The graph illustrates that 125 (76%) women started using reliable contraceptive in the first month of follow-up and 80% used reliable contraceptives at month 6. Overall, 73 (45%) of 164 women used DMPA, and 36 (22%) oral pills.

In the 164 women that were eligible for promotion of reliable contraceptives at baseline, age group and reporting having sex under the influence of alcohol in the month preceding the interview were independently associated with uptake of reliable contraceptives by six months of follow up (in a model adjusted for factors in Table 3). Women aged 18–34 years were twice more likely to take up a reliable method, adjusted odds ratio (aOR) = 2.47, 95%CI: 1.01–6.07 compared to those aged 35 years or more. Those reporting sometimes having sex under influence of alcohol were less likely to take up a reliable method compared to never (aOR = 0.37, 95%CI: 0.14–0.96, Table 3). Though not statistically significant, in this sample, women who had attained some formal education were three times (aOR = 3.21, 95%CI: 0.73–14.16) for primary education and four times (aOR = 4.41, 95%CI: 0.89–21.87) for secondary education more likely to take up a reliable method compared to those without any formal education.

**Table 3:** Proportion and factors associated with uptake of reliable contraceptives among 164 women that were not using reliable methods at enrolled into SiVET in Uganda (2012–2017).

Participant characteristic	FF n (%)	Uptake		FSW n(%)	uOR (95%CI)	LRT p-value	aOR (95%CI)
		FF	FSW				
Overall	53 (100)	111 (100)	39 (73.6)	92 (82.9)	na	na	na
Site							
Masaka	53 (100)	na	39 (73.6)	na	1.00	0.172	1.00
Kampala	na	111 (100)	na	92 (82.9)	1.74 (0.79–3.81)		1.37 (0.50–3.73)
Age group (years)							
35+	16 (30.2)	34 (30.6)	8 (50.0)	27 (79.4)	1.00	0.041	1.00
18–34	37 (69.8)	77 (69.4)	31 (83.8)	65 (84.4)	2.29 (1.04–5.02)		2.47 (1.01–6.07)
Tribe							
Muganda	26 (49.1)	55 (49.6)	22 (84.6)	44 (80.0)	1.00	0.120	
Munyankole	5 (9.4)	13 (11.7)	3 (60.0)	11 (84.6)	0.80 (0.23–2.76)		
Munyarwanda	14 (26.4)	10 (9.0)	7 (50.0)	8 (80.0)	0.38 (0.14–1.03)		
Other	8 (15.1)	33 (29.7)	7 (87.5)	29 (87.9)	1.64 (0.55–4.87)		
Education							
None	4 (7.6)	6 (5.4)	2 (50.0)	4 (66.7)	1.00	0.086	1.00
Primary	35 (66.0)	62 (55.9)	25 (71.4)	50 (80.7)	2.27 (0.59–8.78)		3.21 (0.73–14.16)
Secondary+	14 (26.4)	43 (38.7)	12 (85.7)	38 (88.4)	4.76 (1.07–21.17)		4.41 (0.89–21.87)
Religion							
Christian	37 (69.8)	88 (79.3)	27 (73.0)	73 (83.0)	1.00	0.945	
Muslim	16 (30.2)	23 (20.7)	12 (75.0)	19 (82.6)	0.97 (0.40–2.36)		
Marital status							
Single never married	8 (15.1)	30 (27.0)	7 (87.5)	25 (83.3)	1.00	0.156	1.00
Married	19 (35.8)	7 (6.3)	12 (63.2)	5 (71.4)	0.35 (0.11–1.16)		0.39 (0.10–1.62)
Single ever married	26 (49.1)	74 (66.7)	20 (76.9)	62 (83.8)	0.85 (0.31–2.35)		1.08 (0.35–3.39)
Occupation							
Fishing/related	16 (30.2)	0 (0.0)	11 (68.8)	0 (0.0)	1.00	0.223	
Small scale business	21 (39.6)	2 (1.8)	17 (81.0)	2 (100)	2.16 (0.48–9.77)		
Hotel/Bar/Saloon	9 (17.0)	44 (39.6)	7 (77.8)	33 (75.0)	1.40 (0.41–4.78)		
Sex work	0 (0.0)	64 (57.7)	0 (0.0)	56 (87.5)	3.18 (0.88–11.57)		
Other	7 (13.2)	1 (0.9)	4 (57.1)	1 (100)	0.76 (0.13–4.49)		
Duration lived at current location							
0–1, year	15 (28.3)	20 (18.0)	13 (86.7)	15 (75)	1.00	0.984	
>one year	38 (71.7)	91 (82.0)	26 (68.4)	77 (84.6)	0.99 (0.39–2.52)		
Alcohol consumption (previous one month)							
Non	22 (41.5)	27 (24.3)	17 (77.3)	23 (85.2)	1.00	0.173	
Inconsistent	27 (50.9)	49 (44.1)	21 (77.8)	43 (87.8)	1.20 (0.46–3.10)		
Daily	4 (7.6)	35 (31.6)	1 (25.0)	26 (74.3)	0.51 (0.19–1.37)		
Sex under alcohol influence (previous one month)							
Never	33 (62.3)	40 (36.0)	26 (78.8)	36 (90.0)	1.00	0.343	1.00
Sometimes	14 (26.4)	64 (57.7)	10 (71.4)	49 (76.6)	0.55 (0.24–1.26)		0.37 (0.14–0.96)
Frequently	6 (11.3)	7 (6.3)	3 (50.0)	7 (100)	0.59 (0.14–2.50)		0.70 (0.15–3.34)
Drug use (previous one month)							
No	50 (94.3)	31 (27.9)	36 (72.0)	26 (83.9)	1.00	0.292	
Yes	3 (5.7)	80 (72.1)	3 (100)	66 (82.5)	1.51 (0.70–3.26)		
Genital discharge (previous 3 months)							
Yes	37 (69.8)	34 (30.6)	27 (73.0)	26 (76.5)	1.00	0.146	
No	16 (30.2)	77 (69.4)	12 (75.0)	66 (85.7)	1.77 (0.82–3.81)		
Genital sores/ulcer disease (previous 3 months)							
Yes	29 (54.7)	21 (18.9)	21 (72.4)	15 (71.4)	1.00	0.103	1.00
No	24 (45.3)	90 (81.1)	18 (75.0)	77 (85.6)	1.94 (0.88–4.28)		1.92 (0.78–4.73)
Number of sexual partners (previous 3 months)							
0/1	34 (64.2)	1 (0.9)	26 (76.5)	1 (100)	1.00	0.653	
2+	19 (35.8)	110 (99.1)	13 (68.4)	91 (82.7)	1.23 (0.50–3.04)		
New sexual partner (previous 3 months)							
No	0 (0.0)	10 (9.0)	0 (0.0)	9 (90.0)	1.00	0.374	
Yes	53 (100)	101 (91.0)	39 (73.6)	83 (82.2)	0.42 (0.05–3.47)		
Condom use with the new sexual partner							
Never	32 (60.4)	3 (3.0)	24 (75.0)	3 (100)	1.00	0.721	
Inconsistently	13 (24.5)	24 (23.7)	9 (69.2)	22 (91.7)	1.53 (0.47–5.00)		
Always	8 (15.1)	74 (73.3)	6 (75.0)	58 (78.4)	1.00 (0.39–2.56)		
Away from home ≥3 days/week							
Yes	18 (34.0)	38 (34.2)	16 (88.9)	29 (76.3)	1.00	0.912	
No	35 (66.0)	73 (65.8)	23 (65.7)	63 (86.3)	0.96 (0.43–2.15)		

uOR-Unadjusted odds ratio, aOR-Adjusted odds ratio, CI-Confidence interval, %-percent, na-Not applicable, FF-Fisherfolk, FSW-Female sex workers.

Furthermore, 334 (91%; FF = 63/77 (82%) and FSW = 271/290 (93%)) of 367 women enrolled (including the 203 that were already using a reliable method of contraception at baseline) used a reliable method by the end of trial follow up. Of the 334 women that used a reliable method, 197 (59%) used DMPA. Other methods were oral pills (n = 60; 18%), implant (n = 31; 9%), IUD (n = 7; 2%), and sterilized (n = 2; 0.6%). Thirty-seven women (11%) switched between reliable methods. Of these women, most 19 (51%) switched from DMPA to oral pills. All women (131 new reliable contraceptives users and 203 already using at baseline) reported sustained use of a reliable method throughout the follow up period. Less than one tenth of women (n = 33; 9%; FF = 14 and FSW = 19) did not use any reliable method throughout the study. Of these, seven (FF = 6 and FSW = 1) used condoms and 26 (FF = 8 and FSW = 18) did not use any form of contraception.

In the 367 women enrolled in the trial, overall factors independently associated with use of reliable contraceptives by six month of follow up included; age group, with women aged 18–34 years being thrice more likely to use a reliable method of contraceptives (aOR = 2.86, 95%CI: 1.31–6.24) compared to those aged 35 or more years. Other factors included study site [Kampala, aOR = 3.09, 95%CI: 1.36–6.98) compared to Masaka] and education (borderline) [secondary or more, aOR = 3.06, 95%CI: 0.78–12.02) compared to no education].

## Discussion

We investigated in women of reproductive age in the FF and FSW use of reliable contraceptive methods and associated factors at baseline and at the end of a six-month vaccination schedule in two SiVETs. We found that the proportion of sexually active women using a reliable method at baseline was low, about one in every two women. Promotion and provision of reliable methods by the trial staff increased the proportion of women using a reliable method to over 90% at the end of six months of follow up. The baseline use of reliable methods in these populations was higher than the 35% reported in the general population in Uganda^15^. It was also higher than 5%-28% reported in other HIV prevention trials in West Africa^16,17^ and East Africa and Southern Africa^18^. Contrary to these HIV prevention trials, women enrolled into SiVET were selected based on willingness to delay pregnancy through use of a reliable method of contraceptive during the vaccination period. In SiVET, the majority of women reporting reliable contraceptive use at baseline and at the end of vaccination used injectable DMPA, which is consistent with both national data^15^ and data from concluded HIV prevention trials^12,23^ in Uganda.

At baseline, reliable contraceptive use differed significantly by age group and self-reported illicit drug use. Nationally age^15^ has been associated with contraceptive use. This is also consistent with previous studies in East and Southern Africa^18^ that reported association of effective contraceptives use with age. Contrary to previous studies^33,34^ that showed high contraceptives use among non-drug users, we found a twofold higher use of reliable contraceptives among illicit drug users. Women involved in sex work dominated the SiVET protocol and these have been linked to high illicit drug^35^ and contraceptives use^36^. It is likely that the perceived risk of pregnancy in this category of women is higher than that of the general population and could influence practices.

About half of the women were not using reliable contraceptive methods at baseline but majority started using them mainly within the first month of follow up. By six months, nine in every ten women were using a reliable method of contraceptives. Uptake of reliable contraceptives in women that were not using such methods at baseline was independently associated with age group, education (borderline) and self-reported having had sex under alcohol influence. Similarly, older age and lower educational status are associated with lower contraceptive use nationally^15^. Women that reported sometimes or frequently having sex under influence of alcohol were less likely to use reliable contraceptives. Alcohol use has been linked to impaired decision making in complex situations^37^.

Though women switched between contraceptive methods, it was encouraging that the switches were within reliable methods and women sustained use of these methods throughout follow up. Uptake and sustained use coupled with higher baseline use of reliable methods than that in the general population could have played a role in the relatively lower proportion of pregnancy than that observed in other HIV prevention trials^20^. Seven women got pregnant while using reliable contraceptives in the trial. The four women on injectable DMPA had all delayed an injection by about one month perhaps indicating they were unaware of the need to renew on time. Three women were using oral pills and adherence issues with use of oral pills have been well-documented^11^. In an actual HIV vaccine efficacy trial, these women would have to be withdrawn from the trial. Encouraging women to receive their contraception injection or take their pills on time through phone calls and/or home visits would improve adherence.

The retention in SiVET was higher than the average (75%) reported in observational cohorts^7,29,30^ in these populations. High retention in the trial setting is likely a result of the rigorous participant tracking system employed compared to observational cohorts. Though it took about three months for 5% of the new reliable contraceptive users to get on reliable contraceptives, most had done so within a month of enrolment. In these key populations, it may not be necessary to put women on reliable contraceptives for atleast three months before screening for trial enrolment instead these could happen concurrently. The high retention coupled with high screening-enrolment ratio and high use of reliable contraceptives make women in these key populations attractive to enroll in the future HIV vaccine efficacy and other HIV prevention trials.

A limitation of our trial is that we did not collect data on the reasons for not using reliable or any contraceptives. Furthermore, we did not collect data on reasons for switching between reliable methods. Such reasons would have been instrumental in informing modification of messages on contraceptives use in HIV prevention trials in these key populations. Even though the trials (in FF and FSW) were similar, two different study teams with a somewhat different protocol conducted them. Differences in correlates of reliable contraceptive use may exist by site, but the small sample size prevented site stratification. However, other than occupation and illicit drug use, most of the women characteristics in the two populations were comparable.

The major strength of our trial is that we promoted and provided reliable contraceptives in the context of HIV vaccine efficacy trial, counselled women on the importance of reliable contraceptives use and provided them with a method of their choice.

## Conclusion

In this SiVET, the proportion of women using reliable contraceptives improved from one in every two women at baseline to nine in every 10 women at the end of follow up. The use of reliable methods at baseline was particularly higher among young women and illicit drug users. Promotion and provision of reliable contraceptives to women not using them at baseline improved the proportion using them within the first month of follow up. Uptake of reliable contraceptives increased with increasing education and decreased with increasing age and the frequency of having sex under alcohol influence. All women using reliable methods continued to use them (or another reliable method) throughout follow-up. The sustained use highlights the importance of promoting and providing reliable contraceptives to trial participants in populations where pregnancy could lead to discontinuation of the use of investigational product. We observed a lower proportion of women becoming pregnant during the trial follow up than that reported in the concluded HIV prevention trials in the region. This trial could have filled the unmet need for reliable contraceptives in these populations in term of promotion and provision as well as enhancing accurate contraceptive messaging. It is particularly encouraging that concurrent vaccination and provision of contraceptives was possible in these populations making them good candidates for future HIV vaccine efficacy and other prevention trials.

## Declarations

### Ethics approval and consent to participate

The Uganda Virus Research Institute (UVRI) Research and Ethics Committee (GC/127/12/04/22 and GC127/12/06/01) and the Uganda National Council for Science and Technology (HS364 and HS1584) approved the conduct of the SiVET protocols. The London School of Hygiene and Tropical Medicine Observational/Interventions Research Ethics Committee (LSHTM14588) approved the concept leading to this analysis. Written informed consent/assent was obtained for each participant before enrolment.

### Study methods confirmation

We confirm that all methods in this manuscript were performed in accordance with the relevant guidelines and regulations.

## Data Availability

The MRC/UVRI and LSHTM Uganda Research Unit has a data sharing policy accessible at https://www.mrcuganda.org/publications/data-sharing-policy. The policy summarizes the conditions under which data collected by the Unit can be made available to other bona fide researchers, the way in which such researchers can apply to have access to the data and how data will be made available if an application for data sharing is approved. Should any of the other researchers need to have access to the data from which this manuscript was generated, the processes to access the data are well laid out in the policy. The corresponding and other co-author emails have been provided and could be contacted anytime for further clarifications and/or support to access the data.
