# Publisher Correction: Use of reliable contraceptives and its correlates among women participating in Simulated HIV vaccine efficacy trials in key-populations in Uganda

**DOI:** 10.1038/s41598-019-54660-7

**Published:** 2019-11-29

**Authors:** Andrew Abaasa, Jim Todd, Yunia Mayanja, Matt Price, Patricia E. Fast, Pontiano Kaleebu, Stephen Nash

**Affiliations:** 1 MRC/UVRI & LSHTM Uganda Research Unit, Entebbe, Uganda; 2 London School of Hygiene and Tropical Medicine, London, UK; 3 International AIDS Vaccine Initiative, New York, USA; 4 University of California at San Francisco, Department of Epidemiology and Biostatistics, San Francisco, USA; 5 Pediatric Infectious Diseases, School of Medicine, Stanford University, Palo Alto, California, USA

Correction to: *Scientific Reports* 10.1038/s41598-019-51879-2, published online 28 October 2019

The original version of this Article omitted the first name of the corresponding author Andrew Abaasa, whose name was incorrectly written as Abaasa. This has now been corrected in the PDF and HTML versions of the Article.

